# Life Cycle Assessment as Support Tool for Development of Novel Polyelectrolyte Materials Used for Wastewater Treatment

**DOI:** 10.3390/nano13050840

**Published:** 2023-02-23

**Authors:** George Barjoveanu, Carmen Teodosiu, Irina Morosanu, Ramona Ciobanu, Florin Bucatariu, Marcela Mihai

**Affiliations:** 1 Department of Environmental Engineering and Management, “Gheorghe Asachi” Technical University of Iasi, 73 D. Mangeron Street, 700050 Iasi, Romania; 2 “Petru Poni” Institute of Macromolecular Chemistry, 41A Grigore Ghica Voda Alley, 700487 Iasi, Romania

**Keywords:** one-pot coacervate deposition, layer-by-layer deposition, inorganic/organic composites, life cycle assessment, eco-design

## Abstract

This life cycle assessment (LCA) study focused on comparing the environmental performances of two types of synthesis strategies for polyethyleneimine (PEI) coated silica particles (organic/inorganic composites). The classic layer-by-layer and the new approach (one-pot coacervate deposition) were the two synthesis routes that were tested for cadmium ions removal from aqueous solutions by adsorption in equilibrium conditions. Data from the laboratory scale experiments for materials synthesis, testing, and regeneration, were then fed into a life cycle assessment study so that the types and values of environmental impacts associated with these processes could be calculated. Additionally, three eco-design strategies based on material substitution were investigated. The results point out that the one-pot coacervate synthesis route has considerably lower environmental impacts than the layer-by-layer technique. From an LCA methodology point of view, it is important to consider material technical performances when defining the functional unit. From a wider perspective, this research is important as it demonstrates the usefulness of LCA and scenario analysis as environmental support tools for material developers because they highlight environmental hotspots and point out the environmental improvement possibilities from the very early stages of material development.

## 1. Introduction

The discharge of a wide variety of pollutants into the aquatic environment from anthropogenic activities (i.e., agricultural, industrial, and urban waste) causes many concerns in relation to water resource quality, ecosystems, and human health [1]. Among the various water pollutants, heavy metal ions [2], pharmaceuticals [3,4], pesticides [5], dyes [6], and halogenated flame retardants [7] are part of the priority/emerging pollutants and are characterized by low concentrations (in the ng/L and μg/L range), toxicity, carcinogenic and mutagenic effects, and bio-accumulative behavior. These characteristics can generate significant impacts both on human health and living organisms in the aquatic environment and pose significant challenges for water/wastewater treatment technologies [8]. Numerous water and wastewater treatment processes have been developed and employed for their removal or destruction, for example, membrane processes [9,10,11], ion exchange [12], coagulation and flocculation processes [13], adsorption on different sorbents [14,15] and oxidation processes [16,17]. Due to cost-effectiveness, high efficiency, and ease of operation, adsorption is one of the most attractive and applied technologies worldwide [2,18]. Traditional sorbents, such as activated carbon, zeolites, clays, etc., are often incapable of providing high removal efficiencies of these pollutants due to their relatively low sorption capacity [18]. To improve the sorbent’s performance, nanotechnology is used to shape or modify materials based on carbon, bio-materials, metal oxides, silica, engineered nanomaterials, magnetic and non-magnetic nanoparticles, and nanocomposites [14,19]. These novel materials have unique properties such as high adsorption capacity, large surface area, surface-free energy, stability, selectivity, and reusability [20,21]. 

Many studies have investigated the performance of using composite materials for water and wastewater treatment. For example, Menazea et al. [22] studied the interaction between the chitosan/graphene oxide composite and divalent heavy metals (Ni^2+^, Cu^2+^, As^2+^, Cd^2+,^ and Pb^2+^) as an effective removal from wastewater. The results indicate that graphene improves the stability of chitosan and its adsorption reactivity for heavy metal ions. Senguttuvan et al. [6] prepared a polypyrrole/zeolite nanocomposite by chemical oxidation and obtained good removal efficiency of reactive blue and reactive red from the synthetic solution. 

To enhance their sorption capacity and selectivity, the surface of such sorbents needed to be functionalized with organic or inorganic reagents to provide adsorption sites [20]. For example, the silica microparticles surface was modified with a high number of amino (–NH_2_) and carboxylic (–COOH) groups of polyelectrolytes [23], while magnetite (Fe_3_O_4_) nanoparticles were functionalized with thiol (–SH) and carboxylic (–COOH) groups using meso-2,3-dimercaptosuccinic acid [24]. Depending on the functional groups of nanocomposite sorbents, different interaction mechanisms with pollutants can occur, for example, electrostatics, coordinative bonds, and hydrophobic forces.

With respect to their synthesis, different methods were used to obtain nanocomposite sorbents, which were able to interact with inorganic/organic pollutants based on a combination of their structural durability and functional group availability. Nosike et al. [25] synthesized via one deposition a new mercury sorbent based on a zeolitic imidazolate framework (ZIF-90) assembled onto the poly(acrylic acid) (PAA) capped Fe_3_O_4_ nanoparticles and cysteine. The sorbent exhibited fast kinetics and a good sorption capacity for Hg^2+^. Based on the emulsion templating concept, Semenova et al. [26] developed nanocomposite particles of polyethylenimine (PEI)–silica and investigated the adsorption capacity of copper ions. PEI-silica nanocomposites exhibit a great adsorption capacity for inorganic metals due to a large number of functional groups. Bucatariu et al. [27] fabricated the same type of composite utilizing a layer-by-layer (LbL) technique in which PEI and the PAA or PEI_4_-Cu complex and PAA were alternately deposited and cross-linked with glutaraldehyde (GA) onto silica microparticles. The obtained multilayered composites were used in multiple sorption cycles for copper ions (Cu^2+^) from an aqueous solution. The sorption experiments showed that the amount of loaded Cu^2+^ was dependent on the amount of the organic part, while the kinetics of sorption depended more on the number of functional groups available inside the (PEI)_n_ film. Moreover, some review articles briefly present the nanocomposite sorbents used in water and wastewater remediation [21] and recent developments in the synthesis and characterization of composites based on polyelectrolytes [28]. Alternatively, Khan et al. [29] investigated the usage of green nanosorbents for the removal of pharmaceutical contaminants in water and wastewater systems. 

However, most of these novel nanocomposite sorbents are still being studied on a laboratory scale. Furthermore, their potential environmental performance has not been sufficiently studied. Subsequently, there is a knowledge gap on the production feasibility, environmental performance, emissions, wastes, impacts on human health, and the development guidelines for these innovative materials. The development of these nanomaterials should be in accordance with the principles of *sustainable development* and *green chemistry* (waste prevention, design and use of safer chemicals, use of renewable energy and raw materials, and use of less hazardous chemicals) [8,30]. In order to support these principles and to evaluate environmental performance, life cycle assessments (LCA) could be applied. LCA enables the identification and quantification of the impacts generated on the environment, human health, as well as resources and emissions throughout the life span of a product or service (i.e., from raw material extraction, production, use, and disposal, including recycling and reuse) [31].

A recent study proposed and investigated how scenario-based LCA may be used to forecast potential environmental impacts and sustainability hot spots during the design and synthesis phases of two LbL synthesis routes to develop silica//PEI microparticles [8]. From then, the available literature found only a few LCA studies to evaluate the synthesis of nanocomposite sorbents for water/wastewater remediation. For instance, Lawal et al. [32] investigated the impact that was generated by the synthesis of hexagonal boron nitride-magnetite (Fe_3_O_4_) nanocomposites when used as adsorbents for the removal of Cr(VI) ions from aqueous solution, while Garcia-Gonzalez et al. [33] performed an LCA study to determine the environmental impacts associated with the production of silicate-titanate nanotube chitosan beads, the usage to remove cadmium from wastewater, and for recycling. The studies highlighted that electricity and chemical consumption were the main inputs contributing to the total impact at a laboratory scale production. However, most studies define approaches and strategies for safe design in the early production of innovative materials in the area of nanotechnology through LCA or combining LCA with risk assessment and socio-economic assessment [34,35,36].

The objective of this study is to investigate how LCA can be used to evaluate and compare the environmental performance of two synthesis routes to obtain nano-structured materials containing an inorganic silica core that is coated with cross-linked PEI. The two synthesis strategies are layer-by-layer polymer deposition and one-pot coacervate deposition. Our investigation considers the very early stages of product development and is aimed at identifying, quantifying, and comparing the environmental impacts that may arise from the chemicals, synthesis operations, and processes by means of LCA. Initial product testing (removal of Cd^2+^ ions from synthetic wastewater by adsorption at equilibrium) and sorbent regeneration are also included in the LCA analysis and used to define a comprehensive functional unit that could depict environmental impacts more objectively. Another study objective is to develop and use these early environmental profiles to investigate several scenarios related to the eco-design of these materials. This study brings new insight into how LCA can be used as a material design instrument that enables the evaluation of environmental aspects related to nanostructured material synthesis by comparing two different synthesis routes and evaluating three eco-design criteria.

## 2. Materials, Methods and Methodology

### 2.1. Composite Material Synthesis, Characterization and Testing

**Figure 1:**
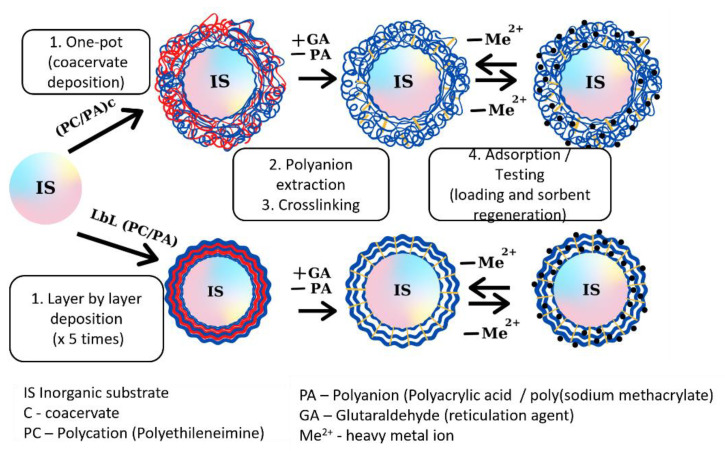
Inorganic/organic composite synthesis routes (adapted with permission after [2]; copyright 2022, Elsevier).

From a technical point of view, the synthesis goals were to obtain organic/inorganic composite materials that would combine the structural stability of inorganic support with a polymeric phase with a high affinity for heavy metal ions and other pollutant species. For this purpose and considering our previous experience [2,8,27,37], the silica microparticles have been used as inorganic support, and where the solid surface was covered (by two different methods) with the polymeric phase: (1) the layer-by-layer deposition of water-soluble PEI and PAA and (2) direct deposition of the organic part using an innovative one-pot interpolyelectrolyte coacervate precipitation, with less material and energy consumption and lack of toxic by-products formation, as presented in Figure 1.

In the layer-by-layer technique, polyelectrolyte films are alternatively deposited on silica microparticles. The procedure consists of introducing the silica core particles (4 g) in 200 mL of a PEI (M_w_ = 25,000 g·mol^−1^) aqueous solution (5·10^−3^ mol·L^−1^, pH = 9.5) for 1 h at room temperature, then by washing them in distilled water to remove the excess polycation and then introducing the newly formed silica/PEI composite in 200 mL of PAA (M_w_ = 10,000 g·mol^−1^) solution (5·10^−3^ mol·L^−1^, pH = 3.5). Finally, particles are thoroughly rinsed again with ultrapure water. This procedure was repeated until nine polymer layers were deposited onto the silica surface, forming the silica//(PEI/PAA)_4.5_ composite.

In the second strategy, the coacervate was generated in situ by the non-stoichiometric combination of PEI, used as polycation, and PAA or with poly(sodium methacrylate) (PMAA) (M_w_ = 1800 g·mol^−1^), used as polyanions.

The second step of the one-pot synthesis through the in situ precipitation of the coacervate is similar to the LbL technique and consists of a chemical cross-linking reaction (in presence of glutaraldehyde, GA) at two different molar ratios between the aldehyde and amino groups, which yielded two cross-linking degrees (r = 0.1 and r = 1). The last synthesis step was the extraction of the unreacted polymeric cations (PEI) and anions (PAA or PMAA) from the cross-linked organic shell in a strongly basic medium.

These materials were tested in single-element sorption experiments, which targeted the removal of Cd^2+^ ions from aqueous solutions [2]. The influence of different parameters was investigated and enabled the estimation of the experimental maximum sorption capacity, as presented in Table 1 [2]. Subsequently, these data were used to calculate the functional unit (mg ions Cd^2+^ removed). It has to be noted that a total of eight nanostructured composites have been obtained by layer-by-layer and four by one-pot synthesis, respectively, but the LCA analysis only includes the materials with the best removal efficiency for Cd^2+^ ions (which are bolded in Table 1).

### 2.2. Life Cycle Assessment Methodology

#### 2.2.1. Goal and Scope Definition

The goal of the LCA analysis was to evaluate the potential environmental impacts caused by the production and use of nano-structured composite materials obtained by LbL and one-pot synthesis routes, which were subsequently used for the removal of Cd^2+^ ions from aqueous solutions. The analysis uses a *cradle-to-gate* approach which includes the following foreground processes: laboratory-scale synthesis processes for inorganic/organic composites and testing (adsorption) processes for the removal of Cd^2+^ ions from synthetic wastewaters. Testing has included repeated sorption/desorption cycles to evaluate the loss of the adsorbent and the evolution of the sorption capacity after repeated cycles. Testing data were used to compute the functional unit for the LCA study. The background system included processes for chemicals and energy production. The analysis did not include transport and disposal processes. Two functional units were defined and used to compare the two synthesis methods, i.e., 1 g of synthesized and purified composites to focus on the synthesis steps, and 1 mg of Cd^2+^ ions were removed to evaluate the heavy metal ion removal efficiency, which is the main functionality of the engineered materials.

#### 2.2.2. Life Cycle Inventory

The life cycle inventory (LCI) data of the inorganic/organic composite materials synthesis was obtained during the laboratory scale experiments, as well as during their testing. The LCI data for the foreground system included primary data generated in the synthesis and testing procedures (repeated cycles of sorption Cd^2+^ ions sorption followed by sorbent regeneration), as presented in Table 2. The background system incorporates the production of electricity (considering the Romanian electricity mix), chemical production, and wastewater treatment, and these data were sourced from the Ecoinvent 3.3 database. As no additional co-products were obtained from the syntheses, all the environmental impacts were allocated to the obtained inorganic/organic composites.

Usually, to compile LCI, databases (e.g., Ecoinvent) are used because they provide easy-to-use data and comprise average data for the large-scale production of chemicals [38]. This, however, is of little help in the case of the particular lab-scale syntheses of highly engineered materials because these use special chemical compounds or special manufacturing synthesis (fine chemicals), which are not usually covered in these databases or are not scope-adequate in terms of their input/output structure or associated environmental impacts. To overcome these uncertainties, some of the inventory entries have been modeled individually in accordance with previous research studies [38,39]. PEI was modeled based on several data available in Eco-invent (ethylenediamine and ring opening polymerization of aziridine); for the poly(acrilic acid), a radical polymerization reaction was modeled based on Zahran et al. [40] and Ristic et al. [41], while the glutaraldehyde was modeled as acetaldehyde. The silica particles used in the synthesis were commercially available, but they were modeled considering a sol-gel co-precipitation reaction protocol [42]. The output flows from these synthesis processes refer to the excess materials which were modeled as waterborne pollutants.

LCI analysis based on the data in Table 1 clearly shows that the one-pot synthesis route significantly uses fewer chemicals (with one magnitude order smaller values). With respect to the quantities used to obtain 1 g of nanocomposites, it may be noticed that the silica core particle accounted for 99% of the particle mass, and the ultrapure water which was used for repeated rinses (in the case of the LbL synthesis) was the chemical with the highest used quantity.

#### 2.2.3. Life Cycle Impact Assessment

The life cycle impact assessment (LCIA) was performed according to the ISO 14040:2006 [43,44] recommendations at the level of classification and characterization steps by using the ReCiPe 2016 method at the midpoint. This LCIA method includes 18 impact categories [45]. SimaPro 9.1.0.11 software was used to compile the inventory and to perform the life cycle impact assessment. LCIA has considered the *cradle-to-gate* approach as previously presented and covered by the inventory description. Current LCIA methodologies do not account for the environmental impacts that are related to nanomaterials release into the environment because there is a lack of characterization factors, fate, and toxicity data related to these compounds.

## 3. Results and Discussion

### 3.1. Environmental Profiles

The two synthesis routes for the composite materials are very similar in terms of the chemicals involved but pretty different considering the operational steps they involve. The layer-by-layer technique comprises a series of successive submersions of particles in solutions and mild agitation and rinsing with ultrapure water, while the one-pot method only employs a single step for the polymeric coating of the inorganic support particle. It is important to note that no electricity or heating is required for any of these synthesis routes. One of the main advantages of these materials is that their synthesis occurs at a normal temperature and that no organic solvents are required, as PEI and PAA are among the water-soluble polymers. Considering the inventory modeling as presented before, the general environmental profiles for two similar particles are presented in Table 3.

The data in Table 3 present the environmental impact values that were obtained by considering each functional unit of the LCA study (1 g composite material obtained and 1 mg Cd^2+^ ions removed). If one compares these environmental profiles on a g/g basis, it may be observed that the two syntheses routes generate similar impact values in most impact categories, with some difference in the freshwater and marine eutrophication and water use categories, where the one-pot synthesis route had less than half of the impacts conducted by the layer-by-layer route. If we refer to absolute impact values, 1 g of the composite material generates about 0.069 kg CO_2_ eq in the climate change category, 0.134 kg 1,4-DCB in the terrestrial eco-toxicity category and 9.1–11.2 L of water in the water use category.

When comparing the composites considering the other functional unit (mg Cd^2+^ ions removed), it is clear that the one-pot synthesis generates considerably lower impacts than the layer-by-layer method; one-pot composites generate only around 24% of the LbL impacts, with the exception of the eutrophication categories, where the impacts are even less (10.9 and 13.3 %).

**Figure 2:**
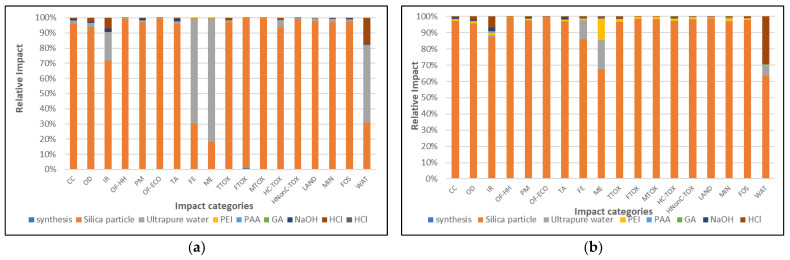
Environmental profile structures of the composite materials (**a**). LbL- IS//(PEI/PAA)_4.5_ (r = 0.1); (**b**) One-pot IS//(PEI/PAA)c (r = 0.1).

In Figure 2, the impact profile structure for obtaining composite materials is presented. The two impact profiles are similar, with the most important contributor being the silica core particle in most of the categories. This is, of course, due to the very high proportion (99%) of the silica core in the overall mass of the composite particles.

**Figure 3:**
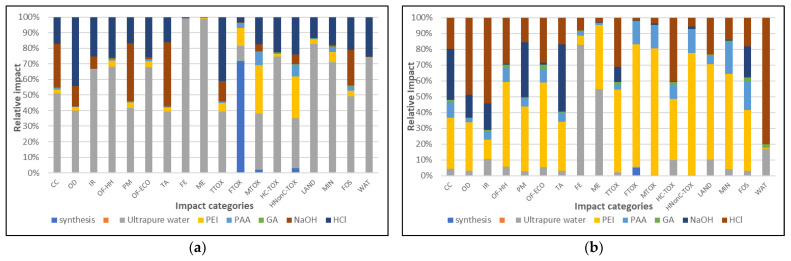
Environmental profile structures of the polymeric particle coating materials (**a**). LbL- IS/(PEI/PAA)_4.5_ (r = 0.1); (**b**) One-pot IS/(PEI/PAA)_c_ (r = 0.1).

To better understand the contribution of other chemicals in the environmental profiles, in Figure 3, only the contribution of the polymeric part of the synthesized materials is displayed.

By analyzing the impact profile structures presented in Figure 3, one may notice that for the composite particles obtained by LbL (Figure 3a), the most important contributor is the use of ultrapure water, which has the highest impact in all categories, except on freshwater toxicity FTOX 9.8%. This is due to the fact that ultrapure water is used in large volumes for dilutions and repeated rinses in the case of LbL synthesis. Ultrapure water impacts are largely due to electricity use for its production and, as presented in Figure 3a, has the highest contribution in the marine (ME) and terrestrial (TE) eutrophication categories (over 99% of the total impact).

In addition to ultrapure water use, hydrochloric acid, and sodium hydroxide are also important contributors in both syntheses. These chemicals are used for the extraction of unreacted polymeric species and have a much higher impact than the actual species involved in obtaining the nanostructured materials, which only have minor contributions to the general impact profile.

In the case of the composites obtained by the one-pot route (Figure 3b), the PEI generated between 5.3 % (WAT) and 79.8% (TE) for most of the categories, making it the most important contributor among the chemicals. The second most important contributor was hydrochloric acid which, similarly to the PEI, generated high impacts in almost all categories. Hydrochloric acid was used as a pH regulator in the single-step synthesis and as an extractor for the unreacted PEI and PAA.

We may notice that the actual synthesis processes had only minor contributions in the toxicity-related categories, and this was due to the wastewater volumes generated after the nanostructured composite particles were rinsed and purified. These impacts account for a maximum of 71.7% for the LbL method and 5.1% for the one-pot route in the freshwater toxicity category (FTOX).

**Figure 4:**
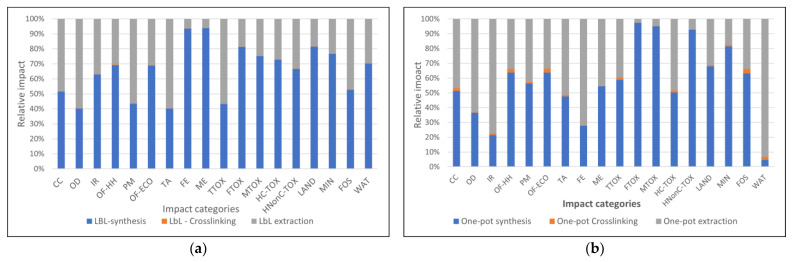
Environmental impacts induced by the synthesis steps (**a**). LbL deposition; (**b**) One-pot synthesis).

By analyzing these environmental profiles, it is possible to identify major contributors to different impact categories. The next step in this analysis would be to identify where changes should be made in the syntheses chain of operations. For this, in Figure 4, the individual contribution of each synthesis step is presented for the layer-by-layer deposition (Figure 4a) and the coacervate deposition (one-pot) synthesis (Figure 4b). These profiles exclude the contribution of the core silica particle because it was clear that these had the most important share in the total impact (Figure 2). The profiles in Figure 4a depict a greater impact of the actual synthesis step in the case of the layer-by-layer deposition method, as compared to the extraction and crosslinking phases. This situation is due to the large quantities of water which were used to rinse the nanostructured composite particles after each layer deposition.

In the case of the one-pot route, there is a more balanced distribution of impacts across categories between the synthesis and extraction process, while the crosslinking demonstrated a slightly higher share (Figure 4b).

### 3.2. Eco-Design Scenarios

The environmental analysis results, which were presented in the previous section, indicate the most important impact contributors that were used as a rationale for studying a series of eco-design scenarios aimed at minimizing the environmental impacts of the one-pot synthesis route. The LbL route has been excluded because it clearly had the worse environmental performance compared to the one-pot method (Table 1). Therefore, focusing on the one-pot reaction route, the scenarios are considered in their potential to replace some of the chemicals in the following sequence.

- Scenario 1: Replacing the silica support particle with inert (quartz) sand.
- Scenario 2: Changing the crosslinking agent concentration (CH_2_ to NH_2_ ratio from 1 to 0.1)
- Scenario 3: Replacing Poly(acrylic acid) with sodium poly(metacrylate).

**Figure 5:**
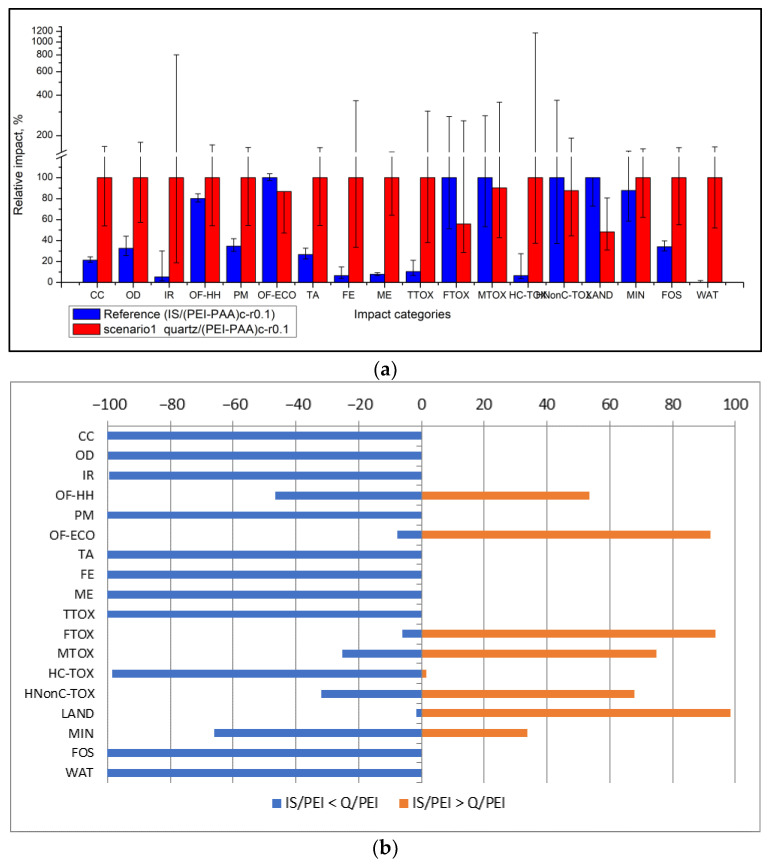
Environmental impacts according to the reference case and Scenario 1 (**a**). Actual impact values (**b**). Probability distribution of differences between the reference case (IS/(PEI/PAA)_c_ (r = 0.1) and Scenario 1 (quartz/(PEI/PAA)_c_ (r = 0.1)).

Scenario 1 considers the replacement of the highly engineered silica support particles with simpler inert quartz particles. To investigate this scenario, the one-pot synthesis route was used to obtain a batch composite quartz/polymeric sorbent starting from a batch of selected sand (70 microns) which was coated with a PEI/PAA polymer in a similar fashion to the silica-cored materials [46]. This composite material was characterized and tested, and it showed very promising results in terms of polymeric content (approx. 3% wt), including very good stability (loss of sorption capacity of approximately 7% after 5 repeated sorption cycles of Cu^2+^ ions), and a good sorption capacity for Cd^2+^ ions (6 mg Cd^2+^ ions/g composite material). Compared to the previous attempts of synthesizing quartz-cored particles through the LbL method [37], these results were very promising from an operational point of view. However, if we compare the environmental impacts generated in scenario 1 with a reference situation given by a correspondent silica core particle, as presented in Figure 5, one may notice that the environmental situation does not improve.

On the contrary, in most categories, the impacts increased because, on the one hand, the inert quartz-cored composite had a considerably lower retention capacity for cadmium ions (albeit much better than the previous LbL attempt), and, on the other hand, the specific impact profile had some high impacts (mainly due to electricity which is used in the initial sieving of the sand particles). Scenario 1 generated slightly lower impacts in the following categories: ozone formation (−14%), freshwater ecotoxicity (−45%), marine ecotoxicity (−10%), human non-carcinogenic toxicity (−13%), and land use (−52%).

It has to be noted that because these profiles are based on laboratory-scaled data, a high degree of uncertainty characterizes these scenario results. Uncertainty was evaluated by means of a Monte Carlo analysis (1000 rounds), which enabled the estimation of confidence intervals for the most likely impact value (usually the mean or median, depending on the fitted probability distribution), as well as some statistic parameters which describe the goodness of approximation. The uncertainty analysis is based on default standard uncertainty values as presented in the EcoInvent 3.3 database for background processes, while for the foreground data (used to compile the life cycle inventories), specific uncertainties were estimated as 10% of the coefficient of variability for each measure. In some cases, greater variability was considered. For example, by considering a uniform distribution of electricity-related variability (e.g., a 0.5, 1.0, or 1.5 kW powered motor for the sieving device), a very high uncertainty was obtained, especially in the impact categories related to electricity use (IR—ionizing radiation, FE—freshwater toxicity and HC_TOX human carcinogenic toxicity), as presented in Figure 5 where uncertainty is depicted as the 95% confidence interval for each impact category. This very high uncertainty hinders a comparison with the reference case, especially where the impact values of the compared situations (including the lower confidence limit) are similar. To overcome this drawback, a comparative Monte Carlo analysis includes that in which the runs estimate the probability that one event is more likely to occur than another based on individual variability data. Such a comparison is presented in Figure 5b, where the blue (negative) represents the probability that the reference case has lower impacts than Scenario 1, and the red bar (positive) represents the probability that the reference case is higher than Scenario 1. It was thus possible to make a clear distinction among the impact categories that were affected by the highest uncertainty: in the IR category, there was only a 0.4% chance that Scenario 1 would have a higher impact than the reference situation, while in the human carcinogenic toxicity category, this probability was 1.4%.

Scenario 2 considers reducing the concentration of the crosslinking agent with a 10-fold factor in an attempt to avoid strong composite cross-linking, which could generate a slightly looser polymeric layer over the core particle. Strong crosslinking is generally associated with the strong packing of polymeric chains, which translates into less accessibility for pollutant species to reach active sorption sites but improves the superficial density of functional groups [28,47]. On the contrary, low composite cross-linking degrees (e.g., 1:10) can improve the accessibility for active functional groups while lessening the stability of the organic layer. The environmental profiles that were presented previously demonstrated that the impact share of the crosslinking agent (glutaraldehyde) was small in all impact categories; therefore, whatever environmental benefit would this change create, it would come from improving the technical performance of the product. This was confirmed experimentally and can be observed in Table 1, where all the maximum sorption capacities increased when the crosslinking concentration increased (when comparing similarly structured materials).

**Figure 6:**
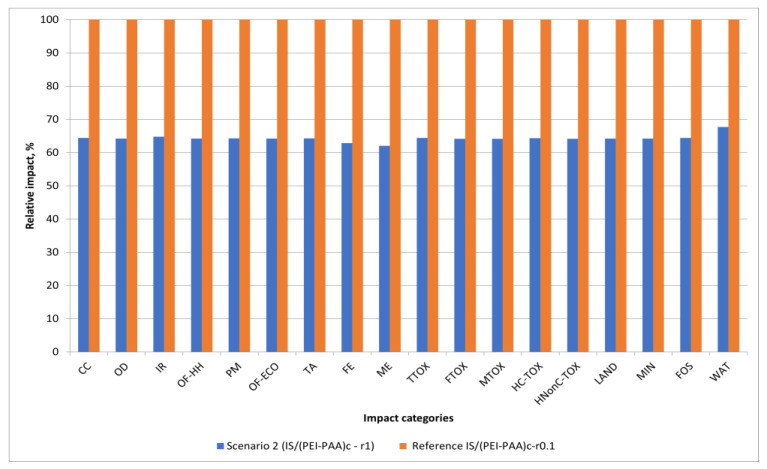
Scenario 2 vs. the reference case comparison of environmental impacts.

In Figure 6, a comparison between a reference case which considers the removal of Cd^2+^ ions through sorption on particles with a 0.1 crosslinking ratio (CH_2_ to NH_2_- concentration), and Scenario 2, which considers the use of the same type of particle, but with a much higher crosslinking ratio (1:1) shows that from an environmental point of view, it is worth cross-linking the polymeric chains at stronger values, as the impacts decrease. By using a more concentrated crosslinking agent (1 M glutaraldehyde), it is possible to decrease environmental impacts by approximately 35% in all categories. This is because, in this case, both situations have similar uncertainties in terms of values, and an uncertainty analysis is not required.

**Figure 7:**
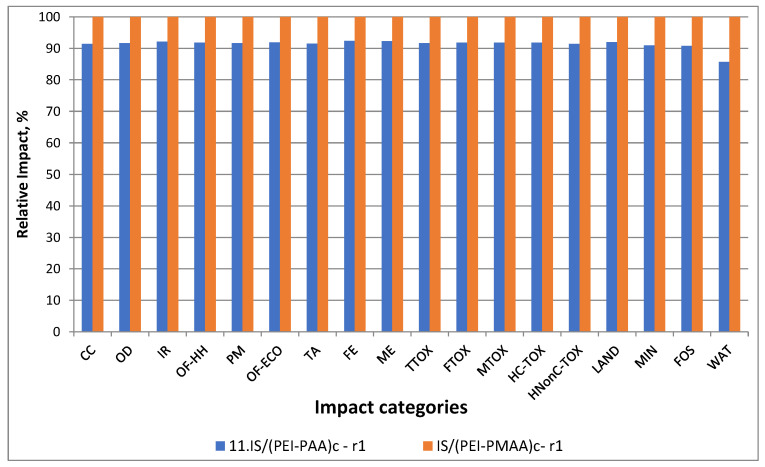
Scenario 3 vs. the reference case comparison of environmental impacts.

In Scenario 3, the replacement of the PAA with PMAA was studied. The results presented in Figure 7 demonstrate that by making this change, it is possible to have lower impact values from 7.91 to 9.26% in all categories, except for water consumption, where this difference was 14.33%.

## 4. Conclusions

This study showcases how life cycle assessment can be used to evaluate the eco-design options for early-stage material development and engineering while allowing the environmental sustainability of novel materials when used for wastewater treatment processes to be evaluated. This LCA study is focused on the comparison of the technical and environmental performances of two types of synthesis strategies for PEI-coated silica particles (organic/inorganic composites), which were tested for Cd^2+^ ion removal from aqueous solutions. Laboratory experiments of materials] synthesis and testing (consisting of repeated cycles of pollutant loading and sorbent regeneration) enabled the identification and quantification of types and values of environmental impacts that were associated with these processes, and three eco-design strategies based on materials substitution were investigated. Concretely, the silica-core particle was found to have the most important contribution to the overall environmental impact profile. The scenario which had considered replacing the highly engineered porous silica core particle with a simpler quartz sand particle did not lead to better environmental performances, despite its promising functional performance. This evaluation demonstrated that the material functionality, as an expression of its technical performance, represented a key aspect in the representation and estimation of the environmental performance of novel materials. From a practical point of view and from the perspective of the material developer, it is important to consider as early as possible in the product development process the eco-design criteria that can evaluate the prospective environmental sustainability of novel materials. At the same time, the LCA results have pointed out that aspects such as functional unit definition, inventory data gaps, data estimation, and associated uncertainties have to be carefully considered and analyzed when interpreting the LCA results. Further research should include the investigation of environmental impacts that are associated with the nano-structured particles released in the environment, which are likely to occur during the use and post-use phases of their life cycle.

## Figures and Tables

**Table 1:** Synthesis routes and experimental data (adapted from [2]).

Synthesis Method	Composite Material	r	Thermogravimetric Analysis		Experimental Data	Isotherm
			Organic Content, %	Calculated Maximum Sorption Capacity, mg Cd^2+^/g Composite	Maximum Sorption Capacity, mg Cd^2+^/g Composite Material	
LbL	IS/(PEI/PAA)_4.5_	0.1	4.2	27.449	16.0	Sips
One-pot	IS/(PEI-PAA)_c_	0.1	10.8	70.583	67	Toth
One-pot	IS/(PEI-PMAA)_c_	0.1	15.7	102.607	69.6	Toth
LbL	IS/(PEI/PAA)_4.5_	1	10.1	66.008	26.67	Toth
One-pot	IS/(PEI-PAA)_c_	1	20.8	135.938	82.8	Toth
One-pot	IS/(PEI-PMAA)_c_	1	15.5	101.300	76.2	Toth
One pot	Q/(PEI/PAA)_c_	0.1	3	19.606	6	Toth

Note: IS—inorganic silica core, Q = quartz sand, PEI—polyethyleneimine, PAA—Polyacrylic acid, PMAA—poly(sodium methacrylate), c—coacervate, r—aldehyde to amino molar ratio.

**Table 2:** Inventory data per 1 g of silica//PEI composites.

No	Inventory Entries		IS/(PEI/PAA)_4.5_ (r = 0.1 LbL)	IS/(PEI-PAA)c (r = 0.1 One Pot)	Comments/Ecoinvent Process
	Composite material	g	1	1	
	Materials/fuels				
1	Silica particle	g	0.99	0.99	SiO_2_ sol gel method
2	Ultrapure water	g	2120	123.6	Water, ultrapure, at plant/GLO U
3	Poly(ethyleneimine)	mg	43	51	Ethylenediamine
4	Poly(acrylic acid)	mg	56	456	PAA-water dispersion (by radical polymerization)
5	Glutaryc anhydride	g	0.0135	0.06	Acetic anhydride from acetaldehyde
7	Sodium hydroxide	g	1.6	1.28	Sodium hydroxide (50% NaOH)
9	Hydrochloric acid	g	0.365	0.292	
	Emissions to water				
10	Amine, tertiary	g	0.01075	0.0102	
11	Glutaraldehyde	g	0.05	0.012	
12	Chemically polluted water	g	162.5	123.6	
13	Acrylic acid	g	0.09	0.0092	

**Table 3:** Environmental impacts of composite silica/PEI materials (per functional unit).

Impact Category	Unit	Symbol	Impacts Per 1 g IS/(PEI/PAA) _4.5_—r = 0.1 (LBL)	Impacts Per 1 g IS/(PEI-PAA)c—r = 0.1 (One-Pot)	Impact Ratio One Pot/Lbl	Impacts Per 1 mg Cd^2+^ Removed byIS/(PEI/PAA)_4.5_—r = 0.1 (LBL)	1 mg Cd^2+^ Removed by IS/(PEI-PAA)c—r = 0.1 (One-Pot)	Impact Ratio One Pot/Lbl
Global warming	kg CO_2_ eq	CC	6.93 × 10^−2^	6.94 × 10^−2^	100.2%	4.33 × 10^−3^	1.04 × 10^−3^	23.9%
Stratospheric ozone depletion	kg CFC11 eq	OD	3.45 × 10^−8^	3.40 × 10^−8^	102.0%	2.07 × 10^−9^	5.05 × 10^−10^	24.4%
Ionizing radiation	kBq Co-60 eq	IR	3.46 × 10^−3^	3.40 × 10^−3^	98.1%	2.16 × 10^−4^	5.07 × 10^−5^	23.4%
Ozone formation, Human health	kg NOx eq	OF-HH	5.21 × 10^−4^	5.20 × 10^−4^	99.9%	3.25 × 10^−5^	7.77 × 10^−6^	23.9%
Fine particulate matter formation	kg PM2.5 eq	PM	1.37 × 10^−4^	1.38 × 10^−4^	100.6%	8.59 × 10^−6^	2.06 × 10^−6^	24.0%
Ozone formation, Terrestrial ecosystems	kg NOx eq	OF-ECO	7.58 × 10^−4^	7.58 × 10^−4^	100.0%	4.74 × 10^−5^	1.13 × 10^−5^	23.9%
Terrestrial acidification	kg SO_2_ eq	TA	2.92 × 10^−4^	2.94 × 10^−4^	100.7%	1.82 × 10^−5^	4.39 × 10^−6^	24.0%
Freshwater eutrophication	kg P eq	FE	5.69 × 10^−5^	3.17 × 10^−5^	55.8%	3.56 × 10^−6^	4.74 × 10^−7^	13.3%
Marine eutrophication	kg N eq	ME	6.14 × 10^−6^	2.81 × 10^−6^	45.7%	3.84 × 10^−7^	4.19 × 10^−8^	10.9%
Terrestrial ecotoxicity	kg 1,4-DCB	TTOX	1.36 × 10^−1^	1.37 × 10^−1^	101.0%	8.48 × 10^−3^	2.05 × 10^−3^	24.1%
Freshwater ecotoxicity	kg 1,4-DCB	FTOX	1.46 × 10^−3^	1.46 × 10^−3^	99.9%	9.11 × 10^−5^	2.17 × 10^−5^	23.8%
Marine ecotoxicity	kg 1,4-DCB	MTOX	2.04 × 10^−3^	2.05 × 10^−3^	100.6%	1.27 × 10^−4^	3.06 × 10^−5^	24.0%
Human carcinogenic toxicity	kg 1,4-DCB	HC-TOX	2.05 × 10^−3^	2.03 × 10^−3^	99.3%	1.28 × 10^−4^	3.03 × 10^−5^	23.7%
Human non-carcinogenic toxicity	kg 1,4-DCB	HNonC-TOX	4.05 × 10^−2^	4.08 × 10^−2^	100.7%	2.53 × 10^−3^	6.08 × 10^−4^	24.0%
Land use	m2a crop eq	LAND	1.97 × 10^−3^	1.96 × 10^−3^	99.7%	1.23 × 10^−4^	2.93 × 10^−5^	23.8%
Mineral resource scarcity	kg Cu eq	MIN	1.10 × 10^−4^	1.10 × 10^−4^	100.2%	6.88 × 10^−6^	1.65 × 10^−6^	23.9%
Fossil resource scarcity	kg oil eq	FOS	2.87 × 10^−2^	2.87 × 10^−2^	100.0%	1.79 × 10^−3^	4.28 × 10^−4^	23.9%
Water consumption	m3	WAT	1.12 × 10^−2^	9.17 × 10^−3^	81.9%	7.00 × 10^−4^	1.37 × 10^−4^	19.6%

## Data Availability

The data presented in this study are available on request from the first author.
